# Combining ^18^F-FDG positron emission tomography with Up-to-seven criteria for selecting suitable liver transplant patients with advanced hepatocellular carcinoma

**DOI:** 10.1038/s41598-017-14430-9

**Published:** 2017-10-26

**Authors:** Arno Kornberg, Ulrike Witt, Martina Schernhammer, Jennifer Kornberg, Gueralp O. Ceyhan, Katharina Mueller, Helmut Friess, Katharina Thrum

**Affiliations:** 1 Department of Surgery, Klinikum rechts der Isar, Technical University, Munich, Germany; 2 Department of Anaesthesiology, Klinikum Großhadern, LMU Munich, Germany; 3 University of Jena, Jena, Germany; 4 Institute of Pathology, Helios Klinikum Berlin, Berlin, Germany

## Abstract

The Up-to-seven (UTS) criteria (sum of tumor size and number not exceeding 7) for indicating liver transplantation (LT) in hepatocellular carcinoma (HCC) were originally based on explant pathology features and absence of microvascular invasion (MVI). ^18^F-fludeoxyglucose (^18^F-FDG) positron emission tomography (PET) was shown to indicate the risk of MVI and tumor recurrence. The aim of this study was to analyze the prognostic significance of the clinical UTS criteria when being combined with PET-status of the tumor. Data of 116 liver transplant patients were subject to retrospective analysis. Five-year recurrence-free survival (RFS) rates in patients meeting (n = 85) and exceeding (n = 21) the radiographic UTS criteria were 81% and 55.1%, respectively (p = 0.014). In the UTS In subset, RFS was significantly better in PET-negative (94.9%) than in PET-positive patients (48.3%; p < 0.001). In the UTS Out subset, 5-year RFS rates were 87.1% and 19% in patients with non- ^18^F-FDG-avid and ^18^F-FDG-avid tumors (p < 0.001), respectively. Positive PET-status was identified as the only independent clinical predictor of tumor recurrence in beyond UTS patients (Hazard ratio [HR] 19.25; p < 0.001). Combining radiographic UTS criteria with FDG-PET may safely expand the HCC selection criteria for LT.

## Introduction

Hepatocellular carcinoma (HCC) is currently the fifth most common cancer and the third most common reason of cancer-related death worldwide^1,2^. Surgical resection is the treatment of choice. However, due to underlying cirrhosis with related liver dysfunction, major hepatectomy is often not practicable^3,4^. From an oncological point of view, liver transplantation (LT) is the best treatment option, since it removes the tumor and the tumor-generating cirrhosis^5,6^. Early experiences with LT for HCC were limited by high recurrence rates and poor survival^7,8^. In a landmark study of 1996, Mazzaferro *et al*. were able to demonstrate that LT in early stage HCC leads to excellent long-term outcome that was not different from patients with non-malignant diseases. Overall (OS) and recurrence-free survival (RFS) rates at 4 years post-LT were 85% and 92% for patients meeting the so-called Milan criteria (MC; one single HCC nodule of up to 5 cm, or a maximum of 3 tumor nodules, each not exceeding 3 cm and absence of macrovascular invasion), but only 50% and 59% for those exceeding them^9,10^. Consequently, the MC were implemented as standard for selecting suitable liver transplant recipients in the major allocation areas around the world.

In recent years, there is a growing concern that the MC may be too restrictive and inappropriate to satisfy continuously growing waiting list demands^11,12^. Several studies have shown that a subset of patients with HCC exceeding standard criteria may benefit from LT. Accordingly, numerous expanded criteria sets were proposed in the last two decades, such as the University of California (UCSF)^13^, Toronto^14^, Valencia^15^ and Hangzhou criteria^16^, to name just a few of them.

In 2009, Mazaferro *et al*. introduced the so-called Up-to-seven (UTS) criteria by simply combining the largest tumor nodule size and the number of HCC nodules, which should not exceed 7 in sum. In a large European multicenter trial including 1556 liver transplant recipients, they demonstrated that patients with HCC exceeding the MC but still meeting the UTS criteria have an excellent prognosis that was not different from patients meeting standard criteria HCC^17^. However, the authors have used post-LT generated histopathologic and not pre-LT radiographic features for their investigation^18^. Apart from that, beneficial outcome of the expanded HCC subset was related to lack of microvascular tumor invasion (MVI)^17^.

Tumor invasion into microscopic vessels is recognized as a major indicator of aggressive tumor behaviour and poor outcome^19,20^. However, it may reliably be assessed only on explant pathology and not by conventional radiographic imaging^21^. Therefore, for a safe application of the UTS criteria, the implementation of appropriate clinical biomarkers of tumor aggressiveness seems to be mandatory^20^.

In recent years, several transplant groups were able to demonstrate that ^18^F-fludeoxyglucose (^18^F-FDG) positron emission tomography (PET) provides useful information on metabolic tumor viability and posttransplant outcome^22–24^. PET-positivity was shown to correlate with presence of unfavourable histopathologic features, like MVI and poor grading^23,25,26^.

The primary aim of this retrospective study was to analyze the prognostic value of the UTS criteria when being based on pretransplant imaging. Apart from that, we investigated whether the combination of the radiographic UTS criteria with ^18^F-FDG PET may be useful for predicting posttransplant tumor recurrence and, thus, for safely expanding the pool of suitable liver transplant patients.

## Methods

### Subjects

The study protocol was approved by the local Ethics Committee (Ethical committee of the Medical School, Technical University Munich, Nr. 217/15). Patients’ registration, waiting list management and transplant procedures were performed according to national law and re-gulations. Prior to LT, all patients gave informed consent that follow-up data may be used for study purpose and respective publication. This work was supported by the German research Foundation (DFG) and the Technische Universität München within the funding programme Open Access Publishing.

From a prospectively updated data base (1996 to 2012; two-center study under same personal responsibilities and conditions), 116 patients that underwent LT for HCC were identified. Tumor diagnosis was established by clinical staging (radiographic imaging by computed tomography [CT] and/or magnetic resonance tomography [MRI] + alpha-fetoprotein level [AFP] measurement) without tumor biopsy. The minimum cut-off tumor nodule size for diagnostic purpose was 1 cm. The MC were primarily used for justifying patients’ listing.

Since December 2007, patients with HCC meeting the MC received exceptional priority status according to the model of end-stage liver disease (MELD) score. Based on HCC topography and remaining liver function, transarterial chemotherapy (TACE) as bridging to LT has been performed. Pretransplant tumor surveillance consisted of liver ultrasound and AFP level determination every 6 weeks, and CT/MRI scan minimum biannually. Additional radiographic imaging was performed when required, such as post-TACE, prior to MELD score upgrading and in the case of tumor-related symptoms.

Progression of tumor load beyond the MC resulted in loss of MELD exceptional priority status. Apart from that, a concise re-evaluation by computed tomography (CT) and/or magnetic resonance tomography (MRI) and AFP level determination every 3 months was initiated. According to an individual decision making process, these patients were primarily scheduled for center-based liver allocation, unless macrovascular tumor infiltration, lymph node infiltration or extrahepatic tumor spread (biological tumor progression) became evident.

^18^F-FDG PET was performed in all patients with liver malignancy in order to exclude extrahepatic tumor manifestation. The prospectively collected data were retrospectively used for the assessment of metabolic tumor properties.

As previously described, we distinguished between PET-positive (PET+ status; ^18^F-FDG-avid) and PET-negative (PET– status; non- ^18^F-FDG-avid) tumors. This classification was based on concise visual FDG-uptake assessment of each tumor nodule in very close morphological demarcation to the surrounding non-tumorous liver regions. Any significantly enhanced ^18^F-FDG uptake pattern compared to normal adjacent liver tissue (tumor to non-tumor maximum standard uptake value > 1) was indicating PET+ status of HCC^22,23^.

Based on final pretransplant radiographic staging, patients were classified as Milan In (HCC meeting the MC; Milan In) or Milan Out (HCC exceeding the MC; Milan Out), and UTS In (HCC meeting the UTS criteria; UTS In) and UTS Out (HCC exceeding the UTS criteria; UTS Out), respectively.

### Transplant procedure and posttransplant follow-up

ABO-compatible deceased donor liver grafts were used for transplant procedure in all study patients. Venous reconstruction was performed by using the piggy back technique without veno-venous bypass. In order to avoid the theoretical risk of systemic tumor cell spread, we have not used intraoperative blood salvage autotransfusion. Posttransplant immunosuppression consisted of a calcineurin inhibitor based regimen either by cyclosporine A or tacrolimus augmented by azathioprine or mycophenolate mofetil. Prednisone was withdrawn latest 3 months post-LT with exception of pre-existing autoimmune hepatitis. Tumor surveillance post-LT consisted of AFP-level determination and liver ultrasound at least every three months. Apart from that, CT scans of the chest and abdomen were performed every 6 months during the first posttransplant year and minimum thereafter or in case of suspected HCC relapse.

### Statistical analysis

Categorical variables were compared using the χ^2^ test. Continuous variables were recorded by median and range, and compared using the Student’s *t* test.

The Kaplan-Meier method was performed to determine overall survival (OS) and recurrence-free survival (RFS) rates. Variables being significant for predicting HCC relapse in univariate analysis (p < 0.05) were entered into a stepwise multivariate logistic Cox regression model in order to identify independent prognostic factors (p < 0.05). Only pretransplant available clinical features were included in the analysis. All statistical analyses were performed by using the software SPSS 23.0 (IBM Inc., Munich, Germany).

## Results

### Clinicopathologic characteristics

The baseline clinicopathologic characteristics are summarized in Table 1.

**Table 1:** Clinicopathologic characteristics of the study cohort (n = 116).

Variable	All patients (n = 116)	UTS In (n = 85)	UTS Out (n = 31)	p value
Gender				0.436
Male	68 (58.6%)	48 (56.5%)	20 (64.5%)	
Female	48 (41.4%)	37 (43.5%)	11 (35.5%)	
Mean age recipients in years ± STD	58.7 ± 6.7	58.8 ± 7.1	58.6 ± 5.7	0.890
Liver disease				0.917
Ethyltoxic	65 (56%)	46 (54.1%)	19 (61.3%)	
Hepatitis C	22 (19%)	18 (21.2%)	4 (12.9%)	
Hepatitis B	12 (10.3%)	9 (10.6%)	3 (9.7%)	
Autoimmun	4 (3.4%)	3 (3.5%)	1 (3.2%)	
Cholestatic	3 (2.6%)	2 (2.3%)	1 (3.2%)	
Other	10 (8.6%)	7 (8.2%)	3 (9.7%)	
Child-Pugh				0.945
A	53 (45.7%)	39 (45.9%)	14 (45.2%)	
B/C	63 (54.3%)	46 (54.1%)	17 (54.8%)	
Median (lab.)MELD score at LT (range)	16 (9–35)	16 (9–35)	18 (9–33)	0.504
Median AFP level in ng/ml at LT (range)	62 (1.5–46930)	50 (1.5–13300)	150 (3.2–46930)	0.111
TACE prior LT				0.456
Yes	76 (65.5%)	54 (63.5%)	22 (71%)	
No	40 (34.5%)	31 (36.5%)	9 (29%)	
HCC nodules*				0.002
Solitary	58 (50%)	50 (58.8%)	8 (25.8%)	
Multifocal	58 (50%)	35 (41.2%)	23 (74.2%)	
Median size of largest HCC nodule in cm (range)*	4 (1–20)	3 (1–6)	6 (3–20)	<0.001
Median total tumor diameter in cm (range)*	5 (1–20)	5 (1–14)	10 (5.8–20)	<0.001
Median tumor number (range)*	1.5 (1–8)	1 (1–5)	3 (1–8)	<0.001
Milan criteria*				<0.001
In	66 (56.9%)	66 (77.6%)	0 (0%)	
Out	50 (43.1%)	19 (22.4%)	31 (100%)	
PET-status				0.076
Negative	75 (64.7%)	59 (69.4%)	16 (51.6%)	
Positive	41 (35.3%)	26 (30.6%)	15 (48.4%)	
Microvascular invasion				0.017
No	73 (62.9%)	59 (69.4%)	14 (45.2%)	
Yes	43 (37.1%)	26 (30.6%)	17 (54.8%)	
Tumor differentiation				0.449
Well/moderate	95 (81.9%)	71 (83.5%)	24 (77.4%)	
Poor	21 (18.1%)	14 (16.5%)	7 (22.6%)	

*According to pretransplant radiographic imaging. ^18^F-FDG – ^18^F-fludeoxyglucose. AFP – alpha-fetoprotein. HCC – hepatocellular carcinoma. LT – liver transplantation. MELD – model for end-stage liver disease. PET – positron emission tomography. TACE – transarterial chemoembolization. UTS – Up-to-seven criteria

Preoperative PET imaging demonstrated 41 PET-positive (35.3%) and 75 PET-negative tumors (64.7%).

Based on clinical staging, 66 patients were classified as Milan In (56.9%) and 50 patients as Milan Out (43.1%), whereas tumors were meeting and exceeding the UTS criteria in 85 (73.3%) and 21 (26.7%) patients, respectively. UTS In and UTS Out patients did not significantly differ with regard to gender, age, liver diseases, Child classification, MELD score, AFP level, TACE and PET status. Explant histopathological studies revealed more tumors demonstrating with MVI in the UTS Out subset (Table 1).

### Overall survival and tumor recurrence

Posttransplant follow-up was ranging between 5 and 184 months (median: 74). OS rates were 82.7% and 75.6% at 3 and 5 years post-LT. Posttransplant HCC relapse was confirmed in 29 patients (25%) after a median of 11 months (range: 4–55).

Corresponding RFS rates were 77.2% and 74.4% at 3 and 5 years, respectively.

In univariate analysis, AFP level, multiple tumor nodules, maximum tumor diameter, number of HCC nodules, UTS criteria and PET-status were significantly associated with risk of HCC recurrence. Only positive PET-status, AFP level > 400 ng/nl and manifestation of multiple HCC nodules were identified as significant and independent predictors of HCC recurrence on multivariate analysis (Table 2).

**Table 2:** Prognostic variables for HCC recurrence in the entire study group (n = 116).

Variable	p value	HR (95% CI)	p value
Male gender	0.663		
Age recipients’ >60 y	0.519		
Viral disease (n)	0.480		
Child B/C	0.162		
(Lab.)MELD >15	0.191		
**AFP prior to LT >400 ng/ml**	**<0.001**	**10.13 (2.341–43.876)**	**0.002**
No TACE pre-LT	0.367		
Multiple HCC nodules*	**0.005**	**4.65 (1.336–16.169)**	**0.016**
Maximum HCC nodule size > 5 cm*	0.226		
Maximum total tumor diameter > 10 cm*	0.038		
Number HCC nodules > 3*	0.013		
Exceeding UTS criteria	0.011		
**PET**+ **status**	**<0.001**	**22.88 (6.303–83.008)**	**<0.001**

*According to pretransplant radiographic staging. AFP – alpha-fetoprotein. CI – confidence interval. HCC – hepatocellular carcinoma. LT – liver transplantation. MELD – model for end-stage liver disease. PET – positron emission tomography. TACE – transarterial chemoembolization. UTS – Up-to-seven criteria

### Outcome according to the Milan and UTS criteria

**Figure 1:**
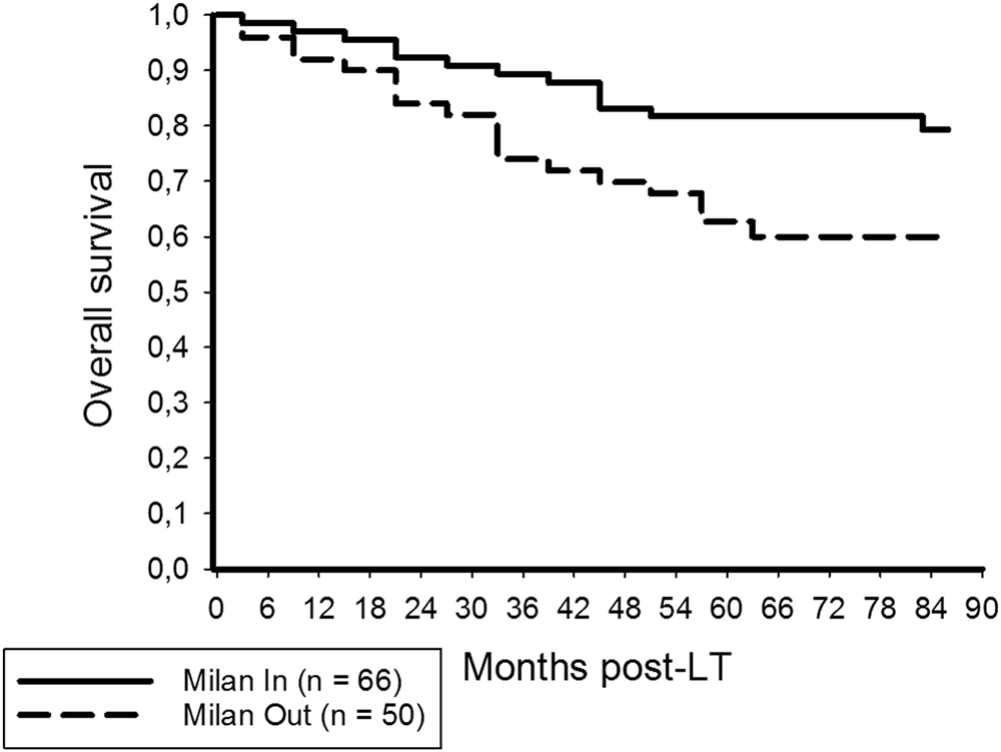
Three and 5-year OS rates among Milan In and Milan Out patients were 89.4% and 81.7%, and 74% and 62.7%, respectively (log rank < 0.001).

**Figure 2:**
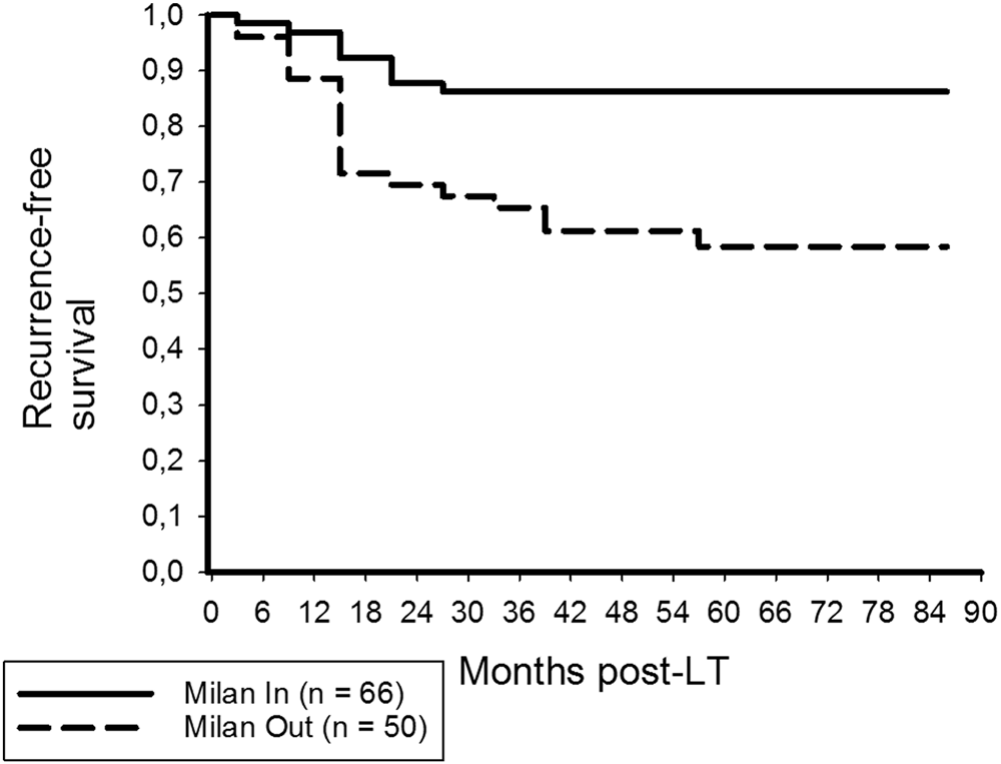
Posttransplant recurrence-free survival rates were 86.2% and 86.2% among Milan In patients, but only 65.4% and 58.4% in patients exceeding the MC (log rank = 0.001).

OS rates among Milan In and Milan Out patients were 89.4% and 81.7%, and 74% and 62.7% at 3 and 5 years post-LT (Fig. 1; p < 0.001). There were 9 HCC recurrences in the Milan In subset (13.6%), whereas 20 Milan Out patients (40%) developed tumor relapse (p = 0.001). RFS rates were 86.2% and 86.2% among Milan In patients, but only 65.4% and 58.4% in patients with Milan Out HCC (Fig. 2; p = 0.001).

**Figure 3:**
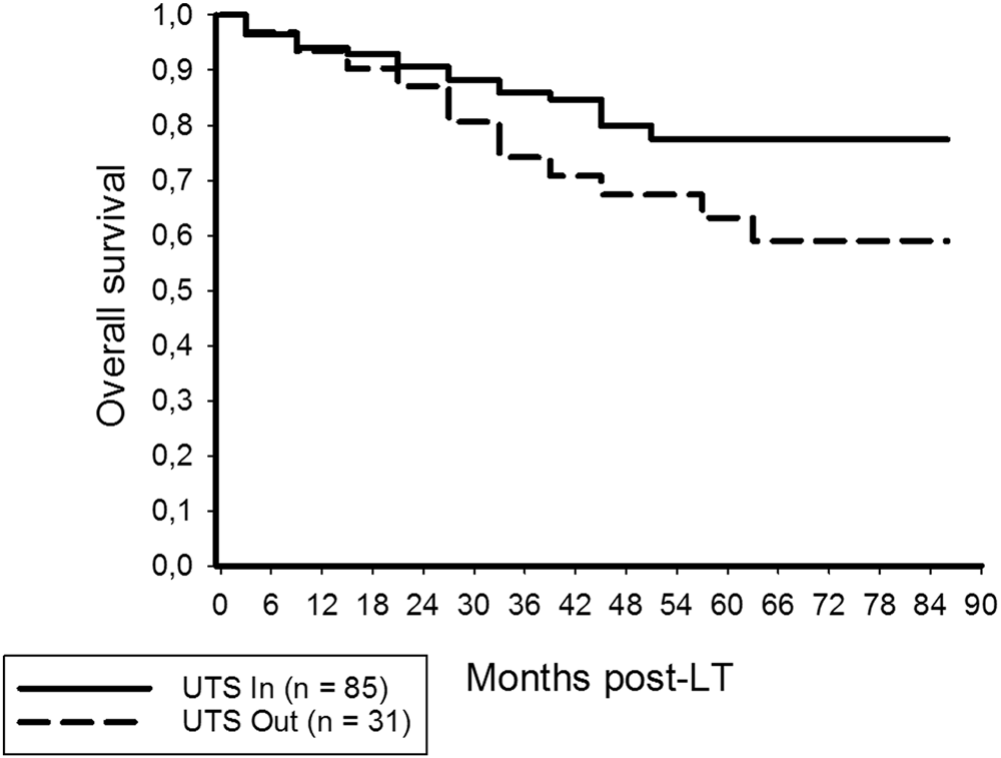
Overall survival at 3- and 5-years post-LT was significantly better in patients meeting the UTS criteria (85.9%, 77.4%) compared to those exceeding them (74.2%, 63.2%; log rank = 0.034).

We found significantly better OS in patients meeting (85.9%, 77.4%) than in those exceeding the UTS criteria (74.2%, 63.2%; p = 0.034; Fig. 3).

Sixteen UTS In (18.8%) and 13 beyond UTS patients (41.9%; p = 0.011) developed HCC recurrence.

**Figure 4:**
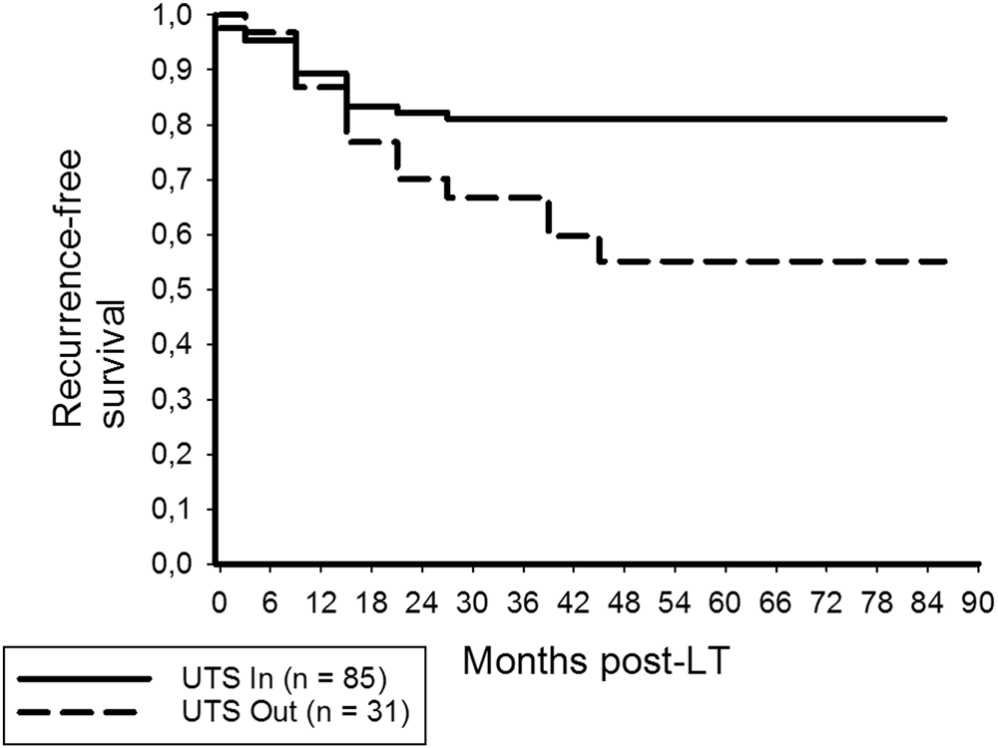
Recurrence-free survival rates at 3- and 5-years post-LT were 81% and 81% in UTS In patients, but only 66.7% and 55.1% in UTS Out patients, respectively (log rank = 0.014).

Thus, RFS rates at 3- and 5-years post-LT were 81% and 81% in patients meeting, but only 66.7% and 55.1% in those exceeding the UTS criteria (p = 0.014; Fig. 4), respectively.

**Figure 5:**
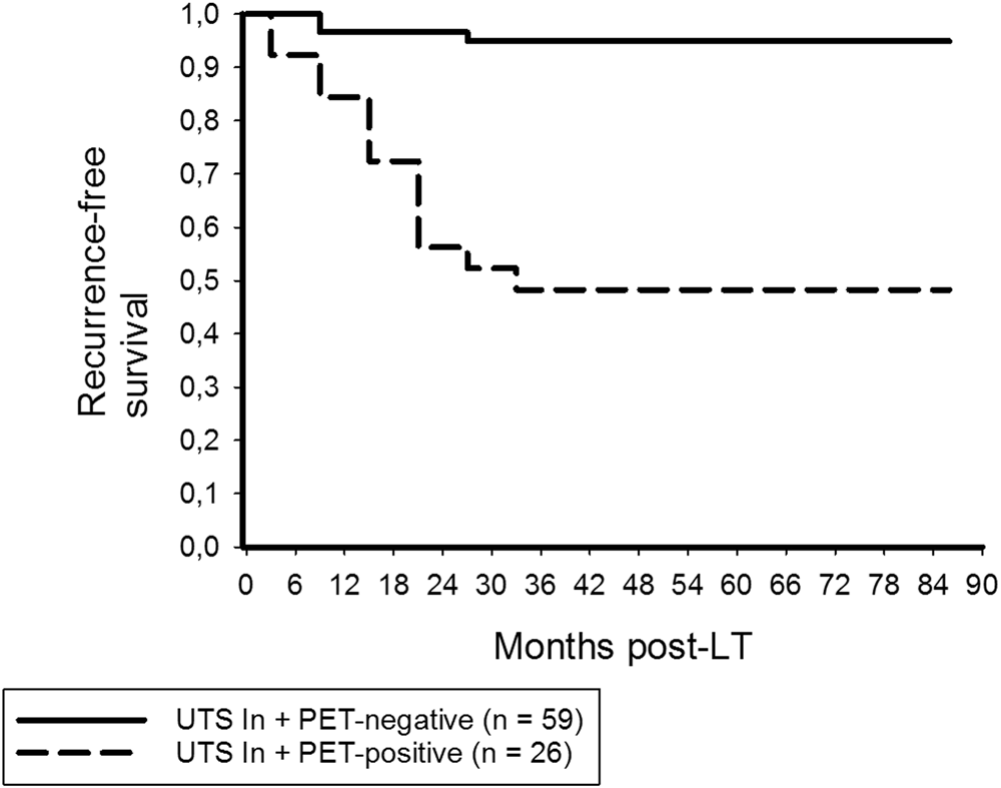
In the UTS In subset, RFS rates at 3- and 5-years post-LT were significantly higher in PET-negative patients (94.9%; 94.9%) as compared to patients with ^18^F-FDG-avid tumors (48.3%; 48.3%; log rank < 0.001).

PET-positivity and presence of more than 3 tumor nodules were identified as the only significant and independent clinical predictors of HCC recurrence among UTS In patients (Table 3). In this subset, RFS was significantly better in PET-negative (94.9%; 94.9%) than in PET-positive patients (48.3%; 48.3%; p < 0.001; Fig. 5).

**Table 3:** Prognostic variables for HCC recurrence in UTS In patients (n = 85).

Variable	p value	HR (95%CI)	p value
Male gender	0.589		
Age recipients’ > 60 y	0.205		
Viral disease (n)	0.215		
Child B/C	0.849		
(Lab.)MELD > 15	0.750		
AFP prior to LT > 400 ng/ml	0.001		
No TACE pre-LT	0.502		
Multiple HCC nodules*	0.013		
Maximum HCC nodule size > 5 cm*	0.945		
Maximum total tumor diameter > 10 cm*	0.491		
Number HCC nodules > 3*	**0.015**	**17.38 (1.443–208.103)**	**0.025**
**PET+ status**	**<0.001**	**24.95 (5.01–124.226)**	**<0.001**

*According to pretransplant radiographic staging. AFP – alpha-fetoprotein. CI – confidence interval. HCC – hepatocellular carcinoma. LT – liver transplantation. MELD – model for end-stage liver disease. PET – positron emission tomography. TACE – transarterial chemoembolization. UTS – Up-to-seven criteria.

**Figure 6:**
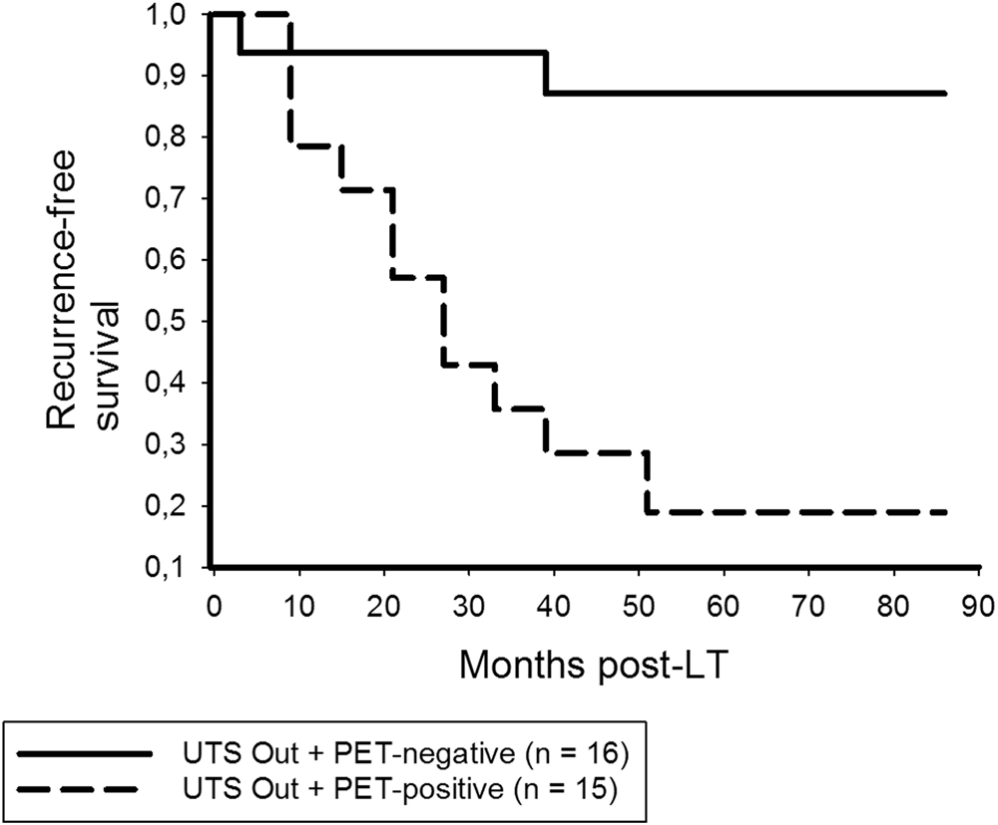
In the UTS Out subset, 3- and 5-year RFS rates were 93.8% and 87.1% in PET-negative, but only 35.7% and 19% in PET-positive patients, respectively (log rank < 0.001).

**Figure 7:**
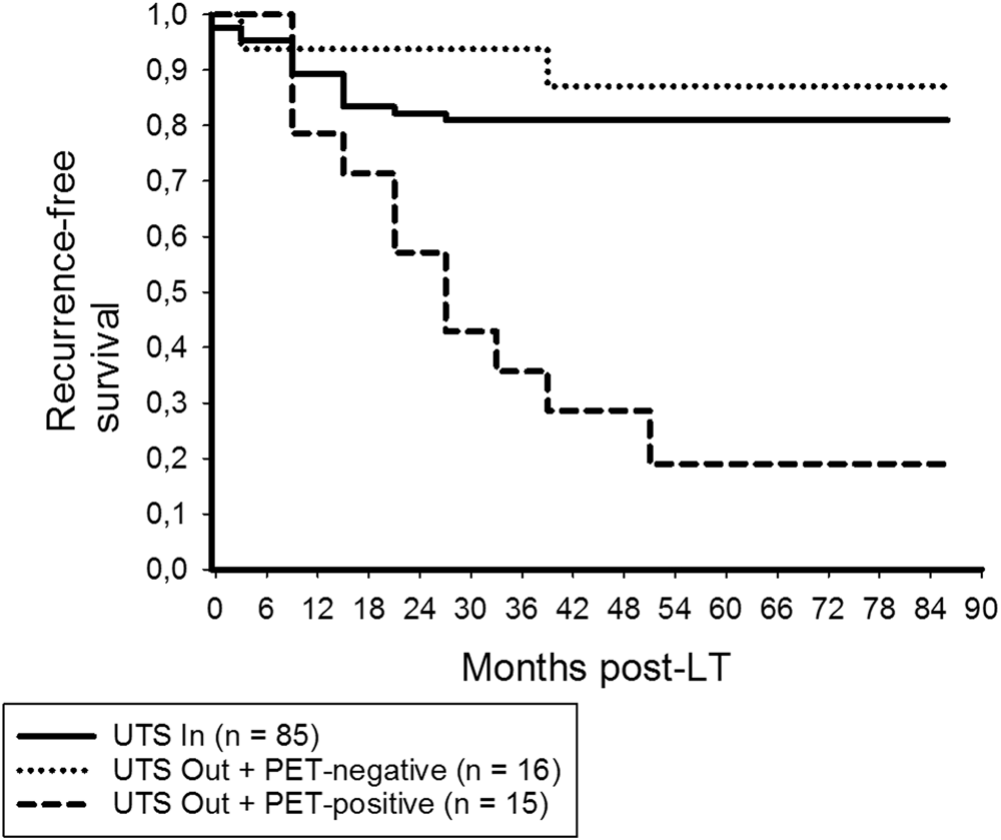
There was no significant difference in RFS between UTS In patients and PET-negative UTS Out patients (log rank = 0.534), whereas it was significantly worse in PET-positive beyond UTS patients (log rank < 0.001).

Only PET-positivity remained as significant and independent predictor of HCC recurrence in beyond UTS patients (Table 4). In this subgroup, we recorded 3- and 5-year RFS rates of 93.8% and 87.1% in non- ^18^F-FDG-avid, but only 35.7% and 19% in ^18^F-FDG-avid patients (p < 0.001; Fig. 6), respectively. RFS was comparable between UTS In and PET-negative beyond UTS patients (p = 0.534; Fig. 7).

**Table 4:** Prognostic variables for HCC recurrence in UTS Out patients (n = 31).

Variable	p value	HR (95%CI)	p value
Male gender	0.768		
Age recipients’ > 60 y	0.739		
Viral disease (n)	0.354		
Child B/C	0.036		
(Lab.)MELD > 20	0.010		
AFP prior to LT > 400 ng/ml	0.028		
No TACE pre-LT	0.326		
Multiple HCC nodules*	0.768		
Maximum HCC nodule size > 5 cm*	0,686		
Maximum total tumor diameter > 10 cm*	0.253		
Number HCC nodules > 3*	0.768		
**PET+ status**	**0.001**	**19.25 (2.961–125.161)**	**0.007**

*According to pretransplant radiographic staging. AFP – alpha-fetoprotein. CI – confidence interval. HCC – hepatocellular carcinoma. LT – liver transplantation. MELD – model for end-stage liver disease. PET – positron emission tomography. TACE – transarterial chemoembolization. UTS – Up-to-seven criteria.

Clinicopathologic differences between Milan In (n = 66), Milan Out/UTS In (n = 19), and beyond UTS patients (n = 31) are listed in Table 5.

**Table 5:** Clinicopathologic differences between Milan In patients (n = 66), Milan Out/UTS In patients (n = 19) and UTS Out patients (n = 31).

	Milan In n = 66	Milan Out/UTS In n = 19	UTS Out n = 31	p value Milan In *vs*. Milan Out/UTS In	p value Milan In *vs*. UTS Out	p value Milan Out/UTS In *vs*. UTS Out
Median AFP prior to LT in ng/ml (range)	45 (1.5–13300)	100 (2.7–5580)	150 (3.2–46930)	0.602	0.144	0.491
TACE pre-LT	42 (63.6%)	12 (63.2%)	22 (71%)	0.970	0.612	0.566
Multiple HCC nodules*	21 (31.8%)	14 (73.7%)	23 (74.2%)	0.001	<0.001	0.968
Median max. HCC nodule size in cm (range)*	3 (1–5)	4.5 (2–6)	6 (3–20)	<0.001	<0.001	0.005
Median total tumor diameter in cm (range)*	4 (1–9)	8 (5.6–14)	10. (5.8–20)	<0.001	<0.001	0.004
Median number HCC nodules (range)*	1 (1–3)	2 (1–5)	3 (1–8)	<0.001	<0.001	0.322
PET+ status	17 (25.8%)	9 (47.4%)	15 (48.4%)	0.072	0.027	0.944
Microvascular invasion	18 (27.3%)	8 (42.1%)	17 (54.8%)	0.216	0.008	0.382
Poor tumor differentiation	9 (13.6%)	5 (26.3%)	7 (22.6%)	0.189	0.268	0.764
HCC recurrence	9 (13.6%)	7 (36.8%)	13 (41.9%)	0.023	0.002	0.721
Overall survival rate				0.001	0.004	0.488
3 year	89.4%	73.7%	74.2%			
5 year	81.7%	62.2%	63.2%			
Recurrence-free survival rate				0.017	0.002	0.837
3 year	86.2%	63.2%	66.7%			
5 year	86.2%	63.2%	55.1%			

*According to pretransplant radiographic staging. AFP – alpha-fetoprotein. HCC – hepatocellular carcinoma. LT – liver transplantation. PET – positron emission tomography. TACE – transarterial chemoembolization. UTS – Up-to-seven criteria.

Tumor recurrence rate was significantly lower, OS and RFS were both significantly better in Milan In patients as compared to the other two subsets. In contrast, there were no significant outcome differences between Milan Out/UTS In patients and patients with beyond UTS tumors (Table 5).

**Figure 8:**
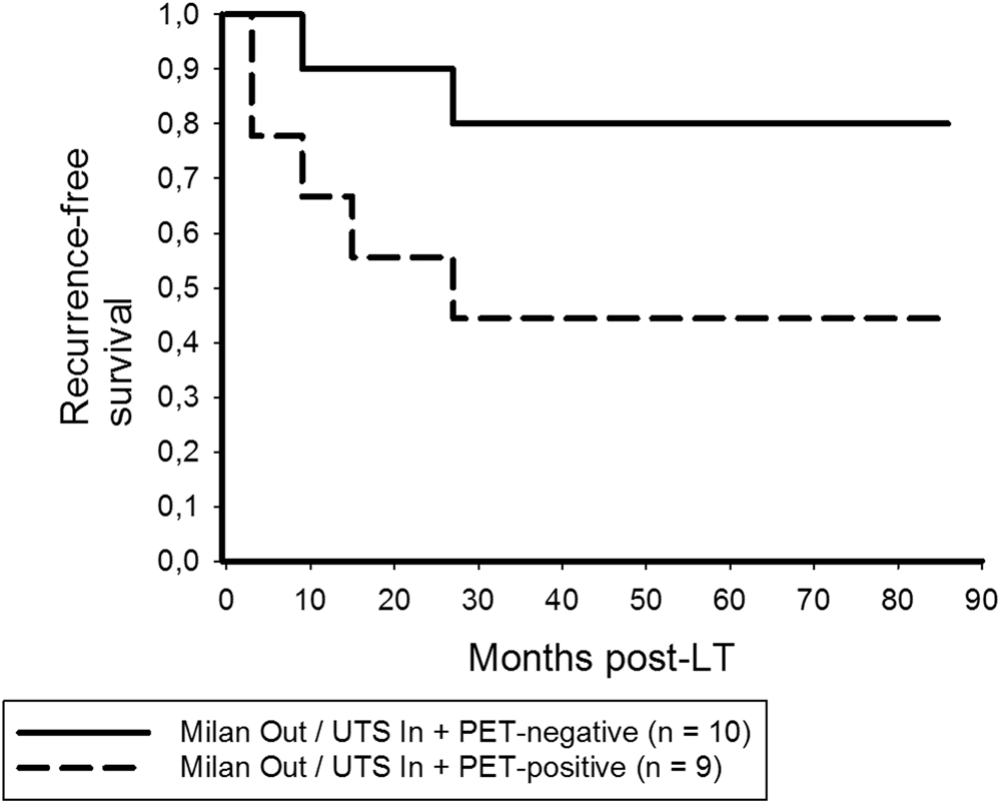
In the Milan Out/UTS In subset, 5-year RFS rates were 80% in PET-positive and 44.4% in PET-negative patients (log rank = 0.078), respectively.

**Figure 9:**
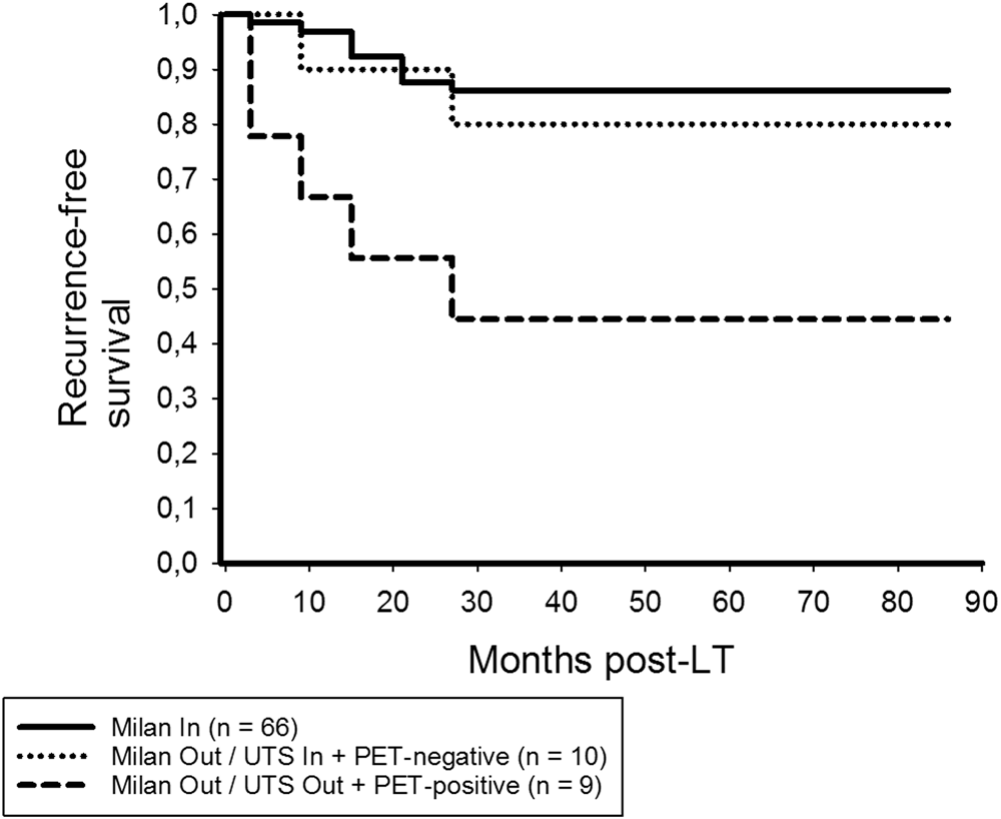
Recurrence-free survival was not significantly different between Milan In patients and those with PET-negative Milan Out/UTS In tumors (log rank = 0.639).

In the Milan Out/UTS In subgroup, 5-year RFS rates were 80% in PET-positive and 44.4% PET-negative patients (p = 0.078; Fig. 8). There was no significant difference in tumor-specific outcome between Milan In patients and patients with PET-negative Milan Out/UTS In tumors (p = 0.639; Fig. 9).

### PET correlations with tumor characteristics and outcome

Table 6 describes the correlations of PET-status with clinicopathologic tumor variables and posttransplant outcome.

**Table 6:** Correlation of FDG-PET with tumor-specific features and outcome.

	PET–(n = 75)	PET + (n = 41)	p value
Median AFP prior to LT in ng/ml (range)	50 (3.2–5580)	146 (1.5–46930)	0.077
Multiple HCC nodules*	34 (45.3%)	24 (58.5%)	0.174
Median max. HCC nodule size in cm (range)*	3 (1–10)	4.6 (1.4–20)	0.005
Median total tumor diameter in cm (range)*	5 (1–16)	8 (1–20)	0.005
Median number HCC nodules (range)*	1 (1–8)	2 (1–6)	0.097
Microvascular invasion			<0.001
no	66 (88%)	7 (17.1%)	
yes	9 (12%)	34 (82.9%)	
Tumor differentiation			0.001
well/moderate	68 (90.7%)	27 (65.9%)	
poor	7 (9.3%)	14 (34.1%)	
HCC recurrence	5 (6.7%)	24 (58.5%)	<0.001
Overall survival rate			<0.001
3 year	97.3%	63.4%	
5 year	88.7%	46.3%	
Recurrence-free survival rate			<0.001
3 year	94.6%	43.8%	
5 year	93.3%	38.1%	

*According to pretransplant radiographic staging. AFP – alpha-fetoprotein. HCC – hepatocellular carcinoma. LT – liver transplantation. PET – positron emission tomography.

Positive PET-status correlated significantly with tumor nodule size and total tumor diameter, whereas it tended to be associated with elevated AFP levels. Apart from that, enhanced ^18^F-FDG uptake on PET was significantly correlated with presence of aggressive histopathologic features (Table 6).

Among PET-negative patients, OS and RFS rates were 97.3% and 88.7%, and 94.6% and 93.3% at 3- and 5-years, which was significantly higher than in PET+ patients (63.4% and 46.3%; 43.8% and 38.1%; p < 0.001). Tumor recurrence rate was 6.7% in non- ^18^F-FDG-avid patients, but 58.5% in those with PET+ tumors (p < 0.001; Table 6).

## Discussion

At first sight, our data seem to implicate that expansion to the clinical UTS criteria does not dramatically increase the risk of tumor recurrence. We found considerable 5year RFS rates in patients meeting the radiographic UTS criteria (81%), which was only slightly inferior to the Milan In cohort (86.2%). Apart from that, an additional of 19 patients beyond the MC could, thereby, be declared as being suitable for LT (Table 5), which corresponds to an increase of 19.6%. Insofar, our study seems to validate the UTS criteria on basis of clinical staging. However, in the additionally recruited patients with tumors exceeding the MC but still meeting the UTS criteria (n = 19), the tumor recurrence rate was significantly higher (36.8% versus 13.6%; p = 0.023), and OS (62.2% versus 81.7%; p = 0.001) and RFS rates (63.2% versus 86.2%; p = 0.017) were both significantly lower compared to the Milan In subset (Table 5). Probably, just because of the small sample size, this has not yet become noticeable in the overall survival. Therefore, on closer examination, our data rather indicate that the undifferentiated application of the UTS criteria enhances the oncological risk.

Whether our results finally justify the implementation of the UTS criteria is a matter of critical discussion. Liberalizing the transplant criteria without expanding the pool of available donor organs results in growing waiting lists and prolongation of pre-LT waiting times. This may in turn enhance the drop out risk following tumor progression. Recently, Volk *et al*. identified a threshold survival of 61% at 5 years to be required for balancing benefit and harm of LT in advanced HCC patients, at least in the US^27^. In our Milan Out/UTS In cohort, 5-year OS and RFS rates were 61% and 63.2% (Table 5), respectively, which exceeded this cut-off value. However, a tumor relapse risk about 40% in an additionally selected expanded criteria HCC subset may be unacceptable, particularly in view of an escalating donor organ shortage^28^.

In recent years, several trials emphasized on the predictive power of the UTS criteria^17,29–31^. Nevertheless, they have not yet been implemented as standard for patients’ selection. It was a major limitation of these studies that pathomorphometric and not radiographic UTS features were used. Well-known discrepancies between pre- and posttransplant tumor staging may have limited clinical applicability^32^. Apart from that, absence of MVI contributed substantially to the prognostic value of the UTS criteria, which additionally hampered their implementation into clinical routine^17,29–31^. In order to create a realistic clinical scenario, we were using only pretransplant available variables in our analysis. Nevertheless, our data indirectly confirmed the specific significance of MVI in this setting, as tumor recurrence rate was significantly higher in the Milan Out/UTS In patients compared to the Milan In subset when MVI was not considered (Table 5).

In fact, vascular tumor invasion is one of the most important prognostic features in LT for HCC^19,20^. While macrovascular infiltration may be appropriately detected by modern radiographic imaging and generally excludes patients from LT, MVI may reliably be confirmed only post-LT^33,34^. Pretransplant tumor biopsy is inappropriate due to high risk of sample errors caused by intratumoral heterogeneity^21^. Apart from that, there is a theoretical risk of tumor cell seeding that might affect posttransplant prognosis^35^.

Since tumor load correlates with risk of MVI and poor grading^36,37^, clinical surrogate markers of tumor aggressiveness are essential for safely expanding macromorphometric selection limits. In the past, response to TACE^38^, AFP-level^39^, C-reactive protein^40^, PIVKA II^41^, yglutamyltransferase^42^ and ^18^F-FDG PET^22,24^ were applied for biological tumor evaluation. Among them, increased ^18^F-FDG uptake on PET was shown to be highly associated with unfavorable histopathology and risk of tumor recurrence^43–45^.

^18^F-FDG PET is a well-established non-invasive tool for metabolic imaging of different malignancies^46^. Like glucose, ^18^F-FDG is uploaded by the tumor cells via several overexpressed glucose transporters. Well differentiated HCC nodules exhibit an enzyme activity that is not different to that of normal liver tissue, resulting in a similar FDG uptake pattern. On contrary, poorly differentiated HCCs are characterized by enhanced FDG uptake pattern as compared to the surrounding healthy liver regions. In contrast to several other cancers, ^18^F-FDG-PET is therefore not appropriate for detection of HCC, but rather for evaluation of metabolic tumor viability^47^.

In fact, the application of ^18^F-FDG PET was recently demonstrated to select suitable liver transplant patients with HCC beyond MC and UCSF criteria^48–51^. Although MVI plays an essential role for the prognostic reliability of the UTS criteria^17,29–31^, the are no comparable investigations in this context.

Our study impressively confirmed that enhanced ^18^F-FDG uptake on pretransplant PET is a valuable indicator of biological tumor aggressiveness and poor outcome (Table 6). Positive PET-status was even identified as most powerful independent clinical predictor of HCC recurrence in our series (Table 2). Apart from that, we were able to demonstrate that combining the clinical UTS criteria with FDG-PET leads to an extremely low tumor relapse risk (Fig. 5). Only 3 of 59 non- ^18^F-FDG-avid (5.1%), but 13 of 26 PET-positive (50%) patients meeting the UTS criteria developed tumor recurrence (p < 0.001).

However, by strictly adhering to this selection concept, more than 30% of our UTS In patients would have been excluded from LT, with almost half of them still being tumor-free alive after 5 years (Fig. 5). The number of liver transplants had thereby been reduced from originally 66 meeting standard criteria (Milan In) to 59 fulfilling the novel hybrid criteria set (UTS In + PET-negative). Rather, our data pointed out that the MC are excellent for selecting suitable liver transplant patients (Figs 1 and 2), whereas further biological tumor evaluation is necessary beyond the Milan boundaries (Table 5).

As shown in Fig. 8, ^18^F-FDG PET identifies those Milan Out/UTS In tumors that have an aggressive biological potential. Probably due to the small sample size (n = 19), the survival difference between PET-positive and PET-negative patients was just not significant (Fig. 8). However, non-^18^F-FDG-avid Milan Out/UTS In patients were able to achieve a 5-year RFS rate of 80%, which was comparable to Milan In patients (86.2%, Fig. 9). This needs to be validated in a larger study cohort.

Noteworthy, we did not find significant outcome differences between Milan Out/UTS In and beyond UTS patients, although tumor load was significantly higher in the UTS Out cohort. One explanation might be that both subgroups did not differ regarding aggressive histopathologic variables (Table 5). According to this finding, the prognostic significance of tumor load may reach a plateau beyond the UTS limit. In fact, none of macromorphometric features but only positive PET-status was identified as an independent clinical promoter of HCC relapse in beyond UTS patients (Table 4).

Furthermore, outcome was not significantly different between patients meeting the UTS criteria and PET-negative patients exceeding them (Fig. 7). This interesting result indicates that biological tumor activity is the major prognostic determinant in advanced HCC stages. D’Amico *et al*. recently identified poor grading and MVI as the only independent predictors of tumor relapse in a series of 124 liver recipients with HCC beyond the UTS criteria, which supports this assumption on a histopathologic basis^31^. If being confirmed in large prospective trials, our data support the implementation of a purely tumor-biology based selection approach, as was already favored by others^52^.

There are several limitations of our study. First, it was a retrospective investigation with all the possible disadvantages of this study design. Second, the number of HCC patients beyond MC but still meeting the UTS criteria was rather low. Another critical point is that we have stratified our data according to semiquantitative and not quantitative PET results, which did not allow for further risk stratification in the ^18^F-FDG-avid subset. In contrast, our study was powered by a large PET data collection and a well-documented long-term follow-up. Apart from that, only preoperatively available tumor characteristics were used for risk analysis.

In conclusion, our study demonstrated that expansion to the clinical UTS criteria carries a considerable risk of selecting tumors with aggressive potential. The implementation of ^18^F-FDG PET improves the prognostic power of the UTS criteria, since it identifies patients with beneficial tumor biology. Expansion to the UTS criteria may, thereby, be safely realized.
